# Impact of the pancreatic enzyme replacement therapy (PERT) shortage on prescribing in England: Analysis of national data

**DOI:** 10.1002/bcp.70703

**Published:** 2026-07-15

**Authors:** Teena S. Varghese, Mary E. Phillips, Louis Fisher, Helen Fitt, Adam Frampton, Christopher Wood, Agnieszka Lemanska

**Affiliations:** ^1^ Faculty of Health and Medical Sciences University of Surrey Guildford UK; ^2^ Nutrition and Dietetics Royal Surrey Hospital NHS Foundation Trust Guildford UK; ^3^ Bennett Institute for Applied Data Science University of Oxford Oxford UK; ^4^ HPB Surgical Unit Royal Surrey NHS Foundation Trust Guildford Surrey UK; ^5^ Imperial Clinical Trials Unit, School of Public Health Imperial College London London UK

**Keywords:** drug shortages, exocrine pancreatic insufficiency, pancreatic enzyme replacement therapy, pancreatic neoplasms, PERT

## Abstract

**Aim:**

Pancreatic enzyme replacement therapy (PERT) is essential for the management of pancreatic exocrine insufficiency. Since June 2023, the United Kingdom has been affected by a global shortage of PERT. We investigated the impact of this shortage on national prescribing trends for Creon, Nutrizym, Pancrex and Pangrol.

**Methods:**

We analysed primary and secondary care prescribing data in England from January 2019 to February 2026 and used linear regression with an interrupted time series approach to assess the impact of the shortage.

**Results:**

Creon 25 000 was the most prescribed PERT with an increasing trend. Following the onset of the shortage, prescribing in primary care increased to 12 192 (95% CI: 11 682–12 702) thousand capsules across 43 203 (41 549–44 856) prescriptions per month but remained below predicted levels (*p* < 0.001). Secondary care prescribing was higher than predicted (*p* < 0.001). Prescribing of Creon 10 000 also increased, with an excess of 912 (493–1330) thousand capsules and 2202 (1308–3097) prescriptions per month in primary care and 235 (192–279) thousand capsules in secondary care (approximately threefold higher than predicted, *p* < 0.001). Nutrizym more than doubled in primary care to 841 (743–940) and tripled in secondary care to 30 (24–35) thousand capsules per month (*p* < 0.001). Pancrex doubled in primary care (*p* = 0.001) but remained below predicted levels in secondary care (*p* = 0.005). The number of capsules per prescription increased for Creon 10 000 by 15 (6–23; *p* = 0.002) but decreased for all other formulations (*p* < 0.001).

**Conclusion:**

Although prescribing remained highest for Creon 25 000, the shortage was associated with a shift towards Creon 10 000 and an increase in alternative products, particularly Nutrizym.

**What is known about this subject**

- Pancreatic enzyme replacement therapy (PERT) is an essential treatment for pancreatic exocrine insufficiency (PEI) in conditions such as pancreatic cancer and cystic fibrosis, improving quality of life and survival.
- The most recent UK‐wide shortage of pancreatin products began in 2023, highlighting persistent vulnerabilities in the PERT supply chain.
- The resulting prescribing and access crisis led to treatment interruptions, product switching and worsening symptoms of PEI.

**What this study adds**

- The shortage substantially altered PERT prescribing patterns, with increased use of Creon 10 000 and reduced prescribing of Creon 25 000 due to limited availability.
- Prescribing of alternative products, including Nutrizym and Pancrex, more than doubled, and switching to lower‐strength formulations increased pill burden.
- Secondary‐care prescribing exceeded expected levels, suggesting adaptations to maintain patient access and prioritization of hospital supply, underscoring the importance of proactive supply management and clear clinical guidance during medicine shortages.

## 1 INTRODUCTION

Pancreatic enzyme replacement therapy (PERT) is an essential treatment for pancreatic exocrine insufficiency (PEI).^1^ PEI is a condition in which the pancreas fails to secrete sufficient digestive enzymes to maintain effective digestive function^2^ and is associated with both pancreatic (primary PEI) and non‐pancreatic causes (secondary PEI).^3^, ^4^ Primary PEI typically results from pancreatic causes, which include acute or chronic pancreatitis, cystic fibrosis, pancreatic cancer and pancreatic resection. Secondary PEI is commonly seen after gastric or bariatric surgery, diabetes mellitus, untreated coeliac disease and inflammatory bowel disease.^5^, ^6^ Clinically, PEI presents with weight loss, diarrhoea, abdominal discomfort, bloating and indigestion due to maldigestion and malabsorption, which can result in reduced quality of life and reduced survival.^2^, ^7^, ^8^ PERT provides exogenous pancreatic enzymes to the duodenum to restore digestion and nutrient absorption and is widely prescribed across both primary and secondary care in the United Kingdom and worldwide.^9^, ^10^, ^11^

The ongoing global disruptions to the production and distribution of pancreatin led to a major shortage, which in the United Kingdom started in late 2023 and resulted in a national prescribing and access crisis.^12^ The shortage affected patient care with substantial challenges to maintaining continuity of therapy.^13^, ^14^, ^15^ Patients and clinicians in both primary and secondary care reported delayed treatment initiation, interrupted supplies and the need to substitute with less familiar or non‐equivalent alternatives. Patients experienced worsening symptoms of maldigestion, weight loss, malnutrition and poor glycaemic control, and, in some cases, admissions to hospital, interruptions to cancer treatment or pancreatic surgery.^16^, ^17^, ^18^ Despite guidance from the Department of Health and Social Care (DHSC) and professional bodies promoting alternative pancreatin preparations, uptake has been inconsistent because of cost considerations, limited prescriber familiarity and uncertainty about dose equivalence between products.^15^

Limited evidence exists on how healthcare services adapted to this ongoing shortage, the workload challenges it has created and the strategies implemented to manage this complex situation. The aim of this study was to assess the impact of the PERT shortage across primary and secondary care in England and to evaluate PERT switching patterns. National prescribing data were analysed to examine trends over time for PERT products, including Creon® (Viatris Pharmaceuticals, UK), the most frequently prescribed formulation, as well as the use of Nutrizym® (Zentiva Pharma, UK), Pancrex® (Essential Pharmaceuticals, UK) and Pangrol® (Target Healthcare, UK [import]), which were relatively less prescribed before the shortage. Regional trends were also assessed to identify differences in prescribing behaviour and local responses. Such analyses can help identify gaps in preparedness, inform policy and support more resilient management of essential medicines such as PERT.

## 2 METHODS

### 2.1 Study design and setting

This was a national study covering 100% of the population in England, conducted across primary and secondary care settings. Analyses were stratified by healthcare setting and seven regions in England: East of England, London, Midlands, North East and Yorkshire, North West, South East and South West.

### 2.2 Study dates

We examined national and regional prescribing trends from January 2019 to February 2026, comparing periods before and after the onset of the PERT shortage in June 2023. Regional trends in primary care were analysed from April 2020 onwards, as the classification of regions in the primary care dataset differed prior to this date and could not be mapped onto the current regional structure.

### 2.3 Data sources

Data were obtained from prescribing datasets published monthly by the NHS Business Services Authority (NHSBSA). The primary care data were extracted from the English Prescribing Dataset (EPD),^19^ which included all general practices in England, and contained prescriptions and quantities of individual medicines dispensed in the community. Secondary care prescribing data were extracted from the Secondary Care Medicines Data (SCMD) dataset,^20^ which contains quantities of individual medicines issued by NHS Trust pharmacy departments in England, including high‐cost and specialist medications.

To validate the findings, we used the OpenPrescribing^21^ and OpenPrescribing Hospitals^22^, ^23^ platforms, hosted by the Bennett Institute for Applied Data Science at the University of Oxford. These platforms allow rapid interrogation and visualization of primary and secondary care prescribing data at multiple administrative levels within the NHS. They were used for exploratory analyses during study design and for verifying results during manuscript preparation.

### 2.4 Outcome measures

We analysed prescribing trends across five outcome measures: (1) Creon 10 000 gastro‐resistant capsules; (2) Creon 25 000 gastro‐resistant capsules; (3) Nutrizym 22 gastro‐resistant capsules; (4) Pancrex and Pancrex V capsules, tablets and oral powder; and (5) Pangrol 10 000 and 25 000 capsules. Data included branded, generic and imported formulations. Creon products were the most commonly prescribed PERT drugs in England, and we assessed switching to alternative PERT products following the onset of the shortage.

Data were curated as monthly capsule counts (in thousands) in primary and secondary care, monthly prescription counts and mean numbers of capsules per prescription in primary care only and regional monthly rates of capsule counts per 100 000 registered patients. Because of the small dispensing quantities, it was not feasible to separate Pancrex and Pangrol by strength or dosage form. For consistency, all quantities were referred to as capsules, including oral powder, which was counted in grammes (reflecting that 1 g of Pancrex V oral powder contained 25 000 units of lipase). Prescription counts and mean numbers of capsules per prescription were not available for the secondary care data because these data represent the issuing of stock rather than prescriptions dispensed to patients. Pangrol was prescribed very sparsely and was therefore presented only in national longitudinal plots and excluded from further statistical analyses.

### 2.5 Statistical analysis

We used descriptive statistics to present monthly prescription and capsule counts as means with 95% confidence intervals (CIs), separately for the periods before and after the onset of the PERT shortage. Linear regression was then applied to data from January 2019 to May 2023 to model trends prior to the shortage and to predict expected trends following its onset (June 2023 to February 2026). Regression coefficients for the trend were reported with 95% CIs and *p*‐values. Observed data were visualized with regression lines in longitudinal plots. To assess the impact of the PERT shortage, we used an interrupted time series approach and reported the difference between observed and predicted values with 95% CIs and *p*‐values. Regional trends were also presented in longitudinal plots as rates per 100 000 registered patients, using GP practice list sizes as the denominator for both data sources. Datasets were downloaded as CSV files and analysed using R (R Core Team, Version 4.5.1, 2025) in RStudio (RStudio Team, Version 2025.05.1).

### 2.6 Ethical considerations

Ethical approval and information governance review were not required because the study used anonymised publicly available data.

## 3 RESULTS

### 3.1 National trends

In primary care during the pre‐shortage period, prescribing of Creon 10 000 showed declining trends for capsules (Figure 1) and prescriptions (Figure 2), decreasing by 24 thousand capsules (95% CI: 16–33; *p* < 0.001) and 88 prescriptions (95% CI: 68–108; *p* < 0.001) per month (Table 1). Creon 25 000 was the most frequently prescribed PERT, with a mean monthly volume of 9126 thousand capsules (95% CI: 8708–9544) dispensed across 33 650 prescriptions (95% CI: 32 386–34 914). Prescribing of Creon 25 000 increased over time, rising by 90 thousand capsules (95% CI: 79–101; *p* < 0.001) and 271 prescriptions (95% CI: 237–305; *p* < 0.001) per month. Nutrizym and Pancrex were prescribed less frequently. Nutrizym had a mean monthly volume of 317 thousand capsules (95% CI: 303–332) across 1000 prescriptions (95% CI: 963–1038), whereas Pancrex had a mean monthly volume of 106 thousand capsules (95% CI: 102–110) across 281 prescriptions (95% CI: 273–288). Pancrex had the highest mean number of capsules per prescription at 378 (95% CI: 372–383). However, this included all formulations with differing lipase strengths: Pancrex V 125 mg capsules (2950 units of lipase per capsule), Pancrex V capsules (8000 units of lipase per capsule) and Pancrex V oral powder (25 000 units of lipase per gram). At 270 (95% CI: 267–273), Creon 25 000 had the lowest number of capsules per prescription. Quantities per prescription increased over time for all products (*p* < 0.001), except for Creon 10 000 (*p* = 0.247).

**FIGURE 1:**
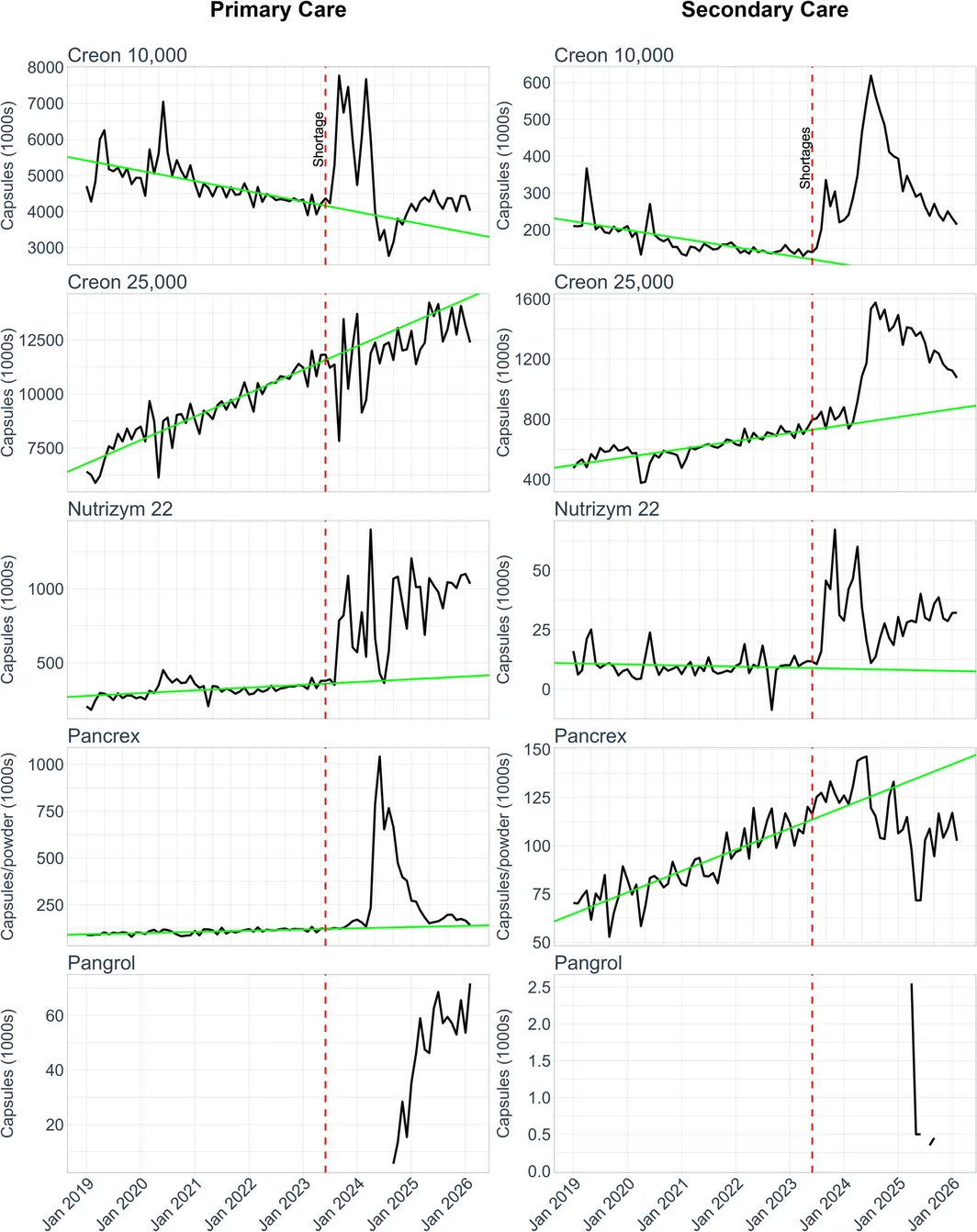
Longitudinal trends in monthly capsule counts (in thousands) in primary care (left) and secondary care (right) for Creon 10 000, Creon 25 000, Nutrizym, Pancrex and Pangrol.

**FIGURE 2:**
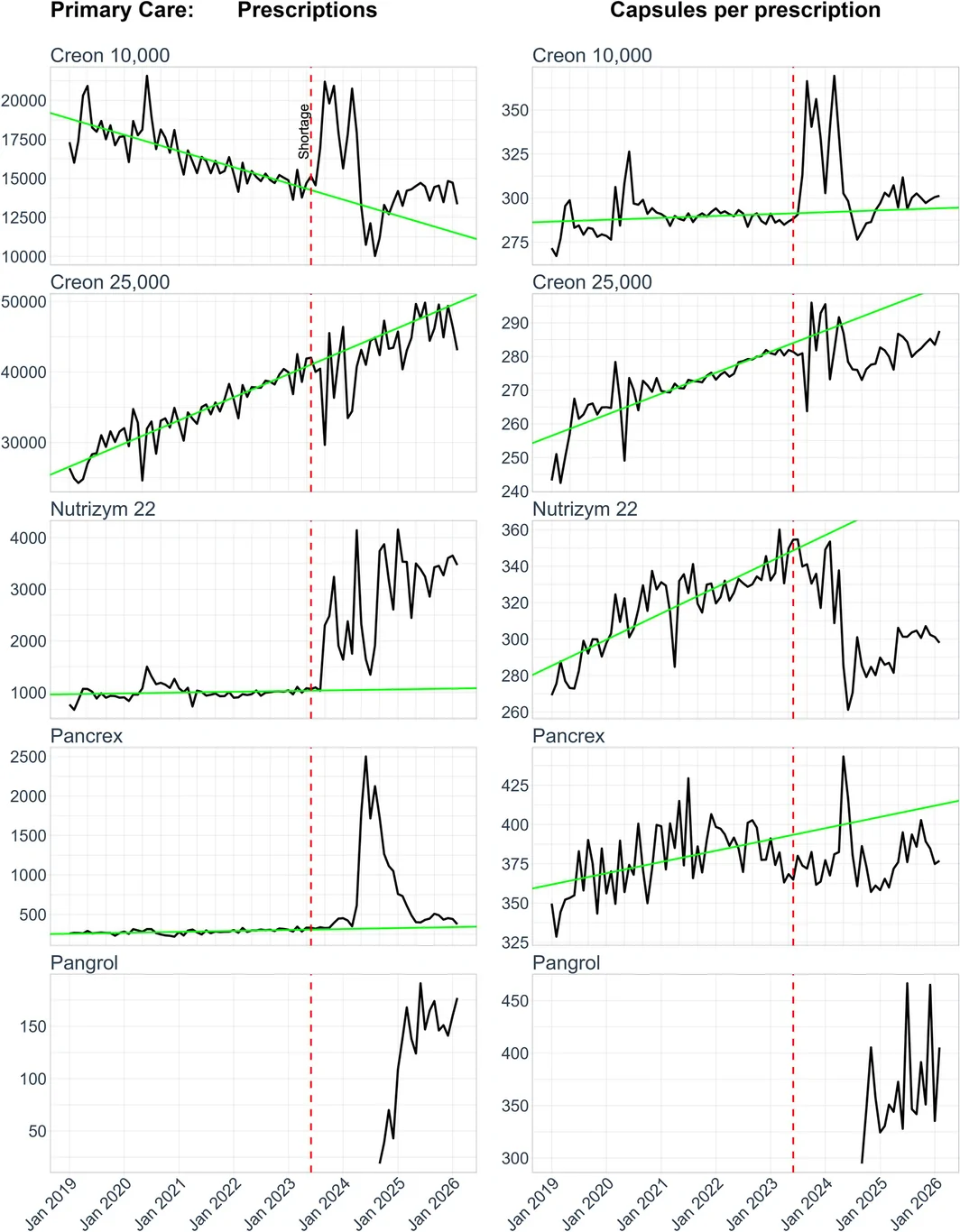
Longitudinal trends in monthly prescription counts and capsules per prescription in primary care for Creon 10 000, Creon 25 000, Nutrizym, Pancrex and Pangrol.

**TABLE 1:** National trends from January 2019 to February 2026 in capsule counts (in thousands) for primary and secondary care, and prescription counts and mean numbers of capsules per prescription for primary care only. Values are presented as monthly means with 95% confidence intervals (CIs) separately for the pre‐shortage period and the period following the onset of the shortage, for Creon 10 000, Creon 25 000, Nutrizym and Pancrex. Trends were modelled using linear regression and interrupted time series analysis, with June 2023 marking the start of the PERT shortage. The shortage effect was estimated as the difference between observed and predicted values.

	Pre‐shortage period (January 2019 to May 2023)			Period following the onset of the shortage (June 2023 to February 2026)			
	Observed	Regression coefficient	*p*‐value	Observed	Predicted	Difference	*p*‐value
Primary care							
Capsules (1000s)							
Creon 10 000	4793 (4630–4956)	−24 (−33 to −16)	<0.001	4667 (4216–5118)	3756 (3673–3838)	912 (493–1330)	<0.001
Creon 25 000	9126 (8708–9544)	90 (79–101)	<0.001	12 192 (11 682–12 702)	12 998 (12 689–13 307)	−806 (−1226 to −387)	<0.001
Nutrizym	317 (303–332)	1 (1–2)	0.001	841 (743–940)	383 (377–388)	458 (363–554)	<0.001
Pancrex	106 (102–110)	1 (0–1)	<0.001	285 (202–369)	129 (127–131)	156 (72–239)	0.001
Prescriptions							
Creon 10 000	16 566 (16 085–17 047)	−88 (−108 to −68)	<0.001	14 968 (13 973–15 963)	12 766 (12 463–13 069)	2202 (1308–3097)	<0.001
Creon 25 000	33 650 (32 386–34 914)	271 (237–305)	<0.001	43 203 (41 549–44 856)	45 307 (44 377–46 236)	−2104 (−3405 to −804)	0.002
Nutrizym	1000 (963–1038)	1 (−1 to 4)	0.317	2765 (2431–3098)	1053 (1049–1057)	1711 (1380–2042)	<0.001
Pancrex	281 (273–288)	1 (0–1)	<0.001	745 (537–952)	321 (318–325)	423 (215–631)	<0.001
Capsules per prescription							
Creon 10 000	289 (286–292)	0 (0–0)	0.247	308 (299–316)	293 (293–293)	15 (6–23)	0.002
Creon 25 000	270 (267–273)	1 (0–1)	<0.001	282 (280–284)	293 (291–294)	−11 (−14 to −8)	<0.001
Nutrizym	316 (310–322)	1 (1–1)	<0.001	307 (298–316)	367 (363–371)	−60 (−72 to −48)	<0.001
Pancrex	378 (372–383)	1 (0–1)	<0.001	379 (372–385)	406 (404–408)	−27 (−34 to −21)	<0.001
Secondary care							
Capsules (1000s)							
Creon 10 000	172 (160–184)	−2 (−3 to −1)	<0.001	321 (278–364)	86 (79–93)	235 (192–279)	<0.001
Creon 25 000	609 (585–633)	4 (3–5)	<0.001	1151 (1054–1247)	793 (778–808)	358 (269–446)	<0.001
Nutrizym	10 (8–11)	0 (0–0)	0.346	30 (26–35)	8 (8–8)	22 (18–27)	<0.001
Pancrex	89 (84–93)	1 (1–1)	<0.001	116 (109–122)	128 (125–131)	−12 (−21 to −4)	0.005

In secondary care during the pre‐shortage period, prescribing was also highest for Creon 25 000, with a mean of 609 thousand capsules per month (95% CI: 585–633) and an increasing trend of 4 thousand capsules per month (95% CI: 3–5; *p* < 0.001). This was followed by Creon 10 000 at 172 thousand capsules per month (95% CI: 160–184), Pancrex at 89 (95% CI: 84–93) and Nutrizym at 10 thousand capsules per month (95% CI: 8–11). Similar to primary care, prescribing of Creon 10 000 declined over time, decreasing by 2 thousand capsules per month (95% CI: 1–3; *p* < 0.001) and prescribing of Creon 25 000 and Pancrex increased over time. Pangrol was rarely prescribed, and prescribing only started from mid‐2025 (Figure 1), and therefore, Pangrol was excluded from the statistical analyses.

### 3.2 Impact of the PERT shortage

Following the onset of the PERT shortage, prescribing patterns in both primary and secondary care changed. In primary care, Creon 10 000, which had previously been declining, rose to a mean of 4667 (95% CI: 4216–5118) thousand capsules per month and 14 968 (95% CI: 13 973–15 963) prescriptions per month. This, compared with predicted values of 3756 (95% CI: 3673–3838) thousand capsules and 12 766 (95% CI: 12 463–13 069) prescriptions, represented an excess of 912 (95% CI: 493–1330; *p* < 0.001) thousand capsules per month and 2202 (95% CI: 1308–3097; *p* < 0.001) prescriptions per month.

In contrast, prescribing of Creon 25 000, which had been increasing during the pre‐shortage period, was lower than expected during the shortage period at 12 192 (95% CI: 11 682–12 702) thousand capsules and 43 203 (95% CI: 41 549–44 856) prescriptions per month. Compared with the predicted 12 998 (95% CI: 12 689–13 307) and 45 307 (95% CI: 44 377–46 236), this represented a reduction of 806 thousand capsules per month (95% CI: 387–1226; *p* < 0.001) and 2104 prescriptions per month (95% CI: 804–3405; *p* = 0.002). The number of capsules per prescription was higher than predicted by 15 (95% CI: 6–23; *p* = 0.002) for Creon 10 000 and lower than predicted by 11 (95% CI: 8–14; *p* < 0.001) for Creon 25 000, averaging at 308 (95% CI: 299–316) capsules per prescription for Creon 10 000 and 282 (95% CI: 280–284) for Creon 25 000. Substitution with alternative products was apparent. Nutrizym prescribing more than doubled during the shortage period, rising to 841 (95% CI: 743–940) thousand capsules per month across 2765 (95% CI: 2431–3098) prescriptions. Compared with the predicted 383 (95% CI: 377–388) and 1053 (95% CI: 1049–1057), this represented an increase of 458 (95% CI: 363–554; *p* < 0.001) thousand capsules per month and 1711 (95% CI: 1380–2042; *p* < 0.001) prescriptions per month. Pancrex prescribing also more than doubled to mean monthly 285 (95% CI: 202–369) thousand capsules across 745 (95% CI: 537–952) prescriptions per month, an increase of 156 (95% CI: 72–239; *p* = 0.001) thousand capsules and 423 (95% CI: 215–631; *p* < 0.001) prescriptions per month. Although the volume of prescriptions and capsules more than doubled, the number of capsules per prescription decreased for both products, by 60 (95% CI: 48–72; *p* < 0.001) capsules per prescription for Nutrizym and 27 (95% CI: 21–34; *p* < 0.001) for Pancrex. In secondary care, prescribing of Creon 10 000 increased after the onset of the shortage to 321 (95% CI: 278–364) thousand capsules per month, which was more than three times the predicted 86 (95% CI: 79–93), and represented an excess of 235 (95% CI: 192–279; *p* < 0.001) thousand capsules per month. At 1151 (95% CI: 1054–1247) thousand capsules per month, Creon 25 000 was also higher than predicted, with an excess of 358 (95% CI: 269–446; *p* < 0.001) thousand capsules per month. Nutrizym increased from a predicted 8 (95% CI: 8–8) to 30 (95% CI: 26–35) thousand capsules per month. Pancrex decreased by 12 thousand capsules per month (95% CI: 4–21; *p* = 0.005) from a predicted 128 (95% CI: 125–131) to observed 116 (95% CI: 109–122).

### 3.3 Trends by region

Across the seven regions, prescribing trends and the impact of the PERT shortage reflected overall national trends. However, differences in prescribing were observed between regions (Figure 3). In primary care, following the onset of the PERT shortage, rates for Creon 10 000 ranged from 4.5 thousand capsules per month per 100 000 patients (95% CI: 4.2–4.9) in London to 11.2 (95% CI: 10.0–12.4) in the North East and Yorkshire (Table 2). Creon 25 000 was the most frequently prescribed formulation, with rates ranging from 11.4 (95% CI: 11.0–11.8) in London to 25.2 (95% CI: 24.3–26.2) in the North West. Compared with predicted values in the absence of PERT supply disruption, Creon 10 000 prescribing was consistently higher across all regions, with the largest increase observed in the North East and Yorkshire: 4.1 thousand capsules per month per 100 000 patients (95% CI: 3.0–5.2; *p* < 0.001). In contrast, Creon 25 000 prescribing was below predicted values in four of the seven regions, with the largest reductions in the North East and Yorkshire (−3.4; 95% CI: −4.5 to −2.4; *p* < 0.001) and the South East (−2.7; 95% CI: −3.5 to −2.0; *p* < 0.001).

**FIGURE 3:**
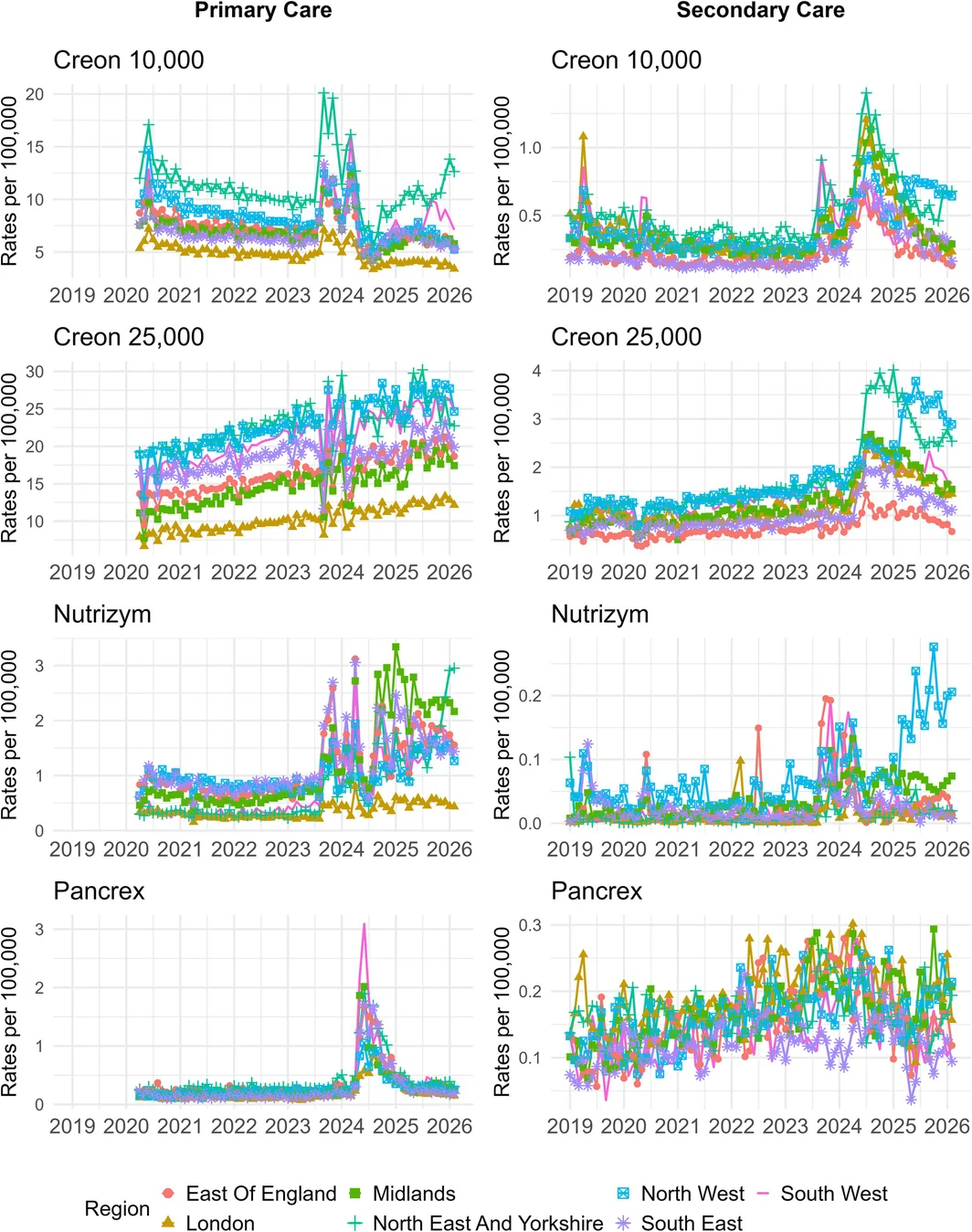
Longitudinal trends by region in monthly capsule counts (in thousands) in primary care (left) and secondary care (right) for Creon 10 000, Creon 25 000, Nutrizym and Pancrex.

**TABLE 2:** Regional trends from June 2023 (following the onset of the PERT shortage) to February 2026 (end of the study) presenting the observed values and differences from the predicted values estimated by the regression model (which would be expected in the absence of the supply disruption). The presented data for primary care and secondary care are rates in monthly capsule counts in thousands per 100 000 registered patients.

Region	Creon 10 000		Creon 25 000		Nutrizym		Pancrex	
	Observed	Difference	Observed	Difference	Observed	Difference	Observed	Difference
Primary care								
East of England	6.8 (6.2–7.4)	1.5 (1.0–2.0), *p* < 0.001	18.0 (17.2–18.8)	−0.6 (−1.2 to 0.1), *p* = 0.076	1.6 (1.4–1.8)	1.1 (0.9–1.3), *p* < 0.001	0.5 (0.4–0.7)	0.3 (0.2–0.5), *p* < 0.001
London	4.5 (4.2–4.9)	1.0 (0.7–1.3), *p* < 0.001	11.4 (11.0–11.8)	0.0 (−0.3 to 0.3), *p* = 0.826	0.5 (0.4–0.5)	0.3 (0.2–0.3), *p* < 0.001	0.3 (0.2–0.4)	0.2 (0.1–0.3), *p* < 0.001
Midlands	6.8 (6.1–7.6)	1.4 (0.7–2.0), *p* < 0.001	16.5 (15.7–17.2)	−1.8 (−2.5 to −1.2), *p* < 0.001	1.9 (1.6–2.2)	1.3 (1.0–1.6), *p* < 0.001	0.5 (0.3–0.6)	0.3 (0.1–0.4), *p* = 0.001
North East and Yorkshire	11.2 (10.0–12.4)	4.1 (3.0–5.2), *p* < 0.001	24.5 (23.4–25.5)	−3.4 (−4.5 to −2.4), *p* < 0.001	1.3 (1.0–1.5)	1.0 (0.7–1.2), *p* < 0.001	0.6 (0.4–0.8)	0.3 (0.1–0.4), *p* = 0.001
North West	7.6 (6.8–8.3)	2.3 (1.7–2.9), *p* < 0.001	25.2 (24.3–26.2)	−0.9 (−1.7 to −0.1), *p* = 0.023	1.2 (1.0–1.3)	0.5 (0.4–0.6), *p* < 0.001	0.4 (0.3–0.5)	0.1 (0.1–0.2), *p* = 0.002
South East	7.0 (6.2–7.8)	2.3 (1.6–3.0), *p* < 0.001	19.5 (18.7–20.4)	−2.7 (−3.5 to −2.0), *p* < 0.001	1.7 (1.5–1.9)	0.9 (0.7–1.1), *p* < 0.001	0.5 (0.3–0.6)	0.4 (0.2–0.5), *p* < 0.001
South West	8.0 (7.1–8.9)	3.3 (2.4–4.1), *p* < 0.001	23.5 (22.5–24.5)	−1.8 (−2.6 to −0.9), *p* < 0.001	1.3 (1.1–1.4)	0.9 (0.7–1.1), *p* < 0.001	0.6 (0.4–0.8)	0.3 (0.0–0.5), *p* = 0.021
Secondary care								
East of England	0.3 (0.3–0.4)	0.2 (0.2–0.3), *p* < 0.001	0.9 (0.9–1.0)	0.2 (0.1–0.2), *p* < 0.001	0.0 (0.0–0.1)	0.0 (0.0–0.0), *p* = 0.012	0.2 (0.2–0.2)	−0.0 (−0.1 to −0.0), *p* = 0.007
London	0.5 (0.4–0.6)	0.5 (0.4–0.6), *p* < 0.001	1.7 (1.5–1.8)	0.6 (0.5–0.8), *p* < 0.001	0.0 (0.0–0.0)	0.0 (0.0–0.0), *p* = 0.006	0.2 (0.2–0.2)	−0.0 (−0.1 to −0.0), *p* = 0.003
Midlands	0.6 (0.5–0.6)	0.4 (0.3–0.5), *p* < 0.001	1.8 (1.7–2.0)	0.6 (0.4–0.7), *p* < 0.001	0.1 (0.1–0.1)	0.0 (0.0–0.0), *p* < 0.001	0.2 (0.2–0.2)	0.0 (−0.0 to 0.0), *p* = 0.905
North East and Yorkshire	0.7 (0.6–0.8)	0.5 (0.4–0.6), *p* < 0.001	2.6 (2.4–2.9)	0.9 (0.6–1.2), *p* < 0.001	0.0 (0.0–0.0)	0.0 (0.0–0.0), *p* < 0.001	0.2 (0.2–0.2)	−0.0 (−0.0 to 0.0), *p* = 0.794
North West	0.6 (0.5–0.7)	0.4 (0.4–0.5), *p* < 0.001	2.5 (2.3–2.7)	0.8 (0.6–1.0), *p* < 0.001	0.1 (0.1–0.1)	0.1 (0.1–0.1), *p* < 0.001	0.2 (0.2–0.2)	−0.0 (−0.0 to −0.0), *p* < 0.001
South East	0.4 (0.3–0.4)	0.3 (0.2–0.4), *p* < 0.001	1.4 (1.2–1.5)	0.4 (0.3–0.6), *p* < 0.001	0.0 (0.0–0.0)	0.0 (0.0–0.0), *p* < 0.001	0.1 (0.1–0.1)	−0.0 (−0.1 to −0.0), *p* < 0.001
South West	0.5 (0.4–0.5)	0.3 (0.3–0.4), *p* < 0.001	1.8 (1.7–2.0)	0.3 (0.2–0.4), *p* < 0.001	0.0 (0.0–0.1)	0.0 (0.0–0.0), *p* = 0.001	0.2 (0.1–0.2)	−0.0 (−0.0 to 0.0), *p* = 0.069

Although Nutrizym and Pancrex accounted for the lowest prescribing quantities, with rates for Nutrizym ranging from 0.5 thousand capsules per month per 100 000 patients (95% CI: 0.4–0.5) in London to 1.9 (95% CI: 1.6–2.2) in the Midlands and rates for Pancrex ranging from 0.3 (95% CI: 0.2–0.3) in London to 0.6 (95% CI: 0.4–0.8) in the North East and Yorkshire and in the East of England, both Nutrizym and Pancrex were above predicted levels in all regions. Nutrizym increases were particularly notable in the Midlands, 1.3 (95% CI: 1.0–1.6; *p* < 0.001), and the East of England, 1.1 (95% CI: 0.9–1.3; *p* < 0.001). The highest increase for Pancrex, 0.4 (95% CI: 0.2–0.5; *p* < 0.001), was observed in the South East, which, relative to the low quantities prescribed before the shortage, represented a sharp but notably transient increase.

In secondary care, Creon 10 000 prescribing ranged from 0.3 thousand capsules per month per 100 000 patients (95% CI: 0.3–0.4) in the East of England to 0.7 (95% CI: 0.6–0.8) in the North East and Yorkshire, with the highest excess over the predicted values in the North East and Yorkshire and in London (0.5; 95% CI: 0.4–0.6; *p* < 0.001). In contrast to primary care, Creon 25 000 prescribing was also consistently higher during the disruption, reaching 2.6 (95% CI: 2.4–2.9) in the North East and Yorkshire, which was 0.9 (95% CI: 0.6–1.2; *p* < 0.001) thousand capsules per month per 100 000 patients above predicted levels.

Observed changes in Nutrizym and Pancrex were generally modest compared with Creon. Although statistically significant differences were noted in some regions, the absolute quantities prescribed were relatively low.

Overall, these findings suggest that while absolute prescribing quantities remained highest for Creon 25 000, the shortage was associated with a shift across all regions towards greater use of Creon 10 000 and modest increases in alternative products, particularly Nutrizym in primary care and to a lesser extent Pancrex, which showed a sharp but transient increase. Importantly, in primary care the shift towards greater use of Creon 10 000 was offset by reductions in Creon 25 000, whereas in secondary care, the shortage was associated with increases in both Creon 10 000 and Creon 25 000 prescriptions.

## 4 DISCUSSION

### 4.1 Summary of findings

The shortage led to dramatic changes in PERT prescribing patterns. In primary care, the previously declining use of Creon 10 000 reversed its downward trend, whereas growth in Creon 25 000 slowed. At the same time, prescribing of alternative products such as Nutrizym and Pancrex increased sharply, more than doubling after the onset of the shortage. This shift resulted in a considerable increase in pill burden for patients, as lower‐strength formulations require approximately 2.5–5 times more capsules to achieve equivalent dosing, depending on which product was prescribed. Increased pill burden is associated with reduced treatment adherence, poorer quality of life and increased treatment‐related stress.^24^ Furthermore, during medicine shortages, patients may miss doses or adjust their prescribed regimens inappropriately, leading to suboptimal symptom control and adverse health outcomes.^25^ The impact may be particularly pronounced among older patients and those managing multiple medications.

In secondary care, prescribing of both Creon formulations and Nutrizym exceeded predicted levels. These findings likely reflected manufacturers' decisions to prioritize hospital supply and the need for rapid adaptation within the healthcare system to maintain patient access. This highlights the importance of proactive supply management and clear clinical guidance during medicine shortages. Timely adaptations can mitigate patient harm and help preserve continuity of essential treatments.

Several integrated care boards (ICBs) implemented local arrangements to support patient access, including allowing secondary care to supply PERT on request from primary care.^26^, ^27^ In other ICBs, hospitals issued ‘rescue prescriptions’ at the request of patients. Although this approach was endorsed by early clinical position statements,^15^ it resulted in an increased workload in secondary care. In addition, the limited availability of Creon resulted in primary care physicians and pharmacists needing to prescribe Nutrizym, Pancrex and later Pangrol, products they were less likely to be familiar with. The variation in regional responses to the shortage observed in this study highlighted the need for a review of mitigation strategies to identify best practices and assess their impact on prescribing patterns and patient access. A systematic review is currently underway to evaluate local policies alongside a more granular analysis of prescribing trends at the ICB level.

### 4.2 Strengths and limitations

The national‐level coverage of the dataset across both primary and secondary care was a key strength of this study. The primary care dataset included all GP practices in England (excluding only prisons and private practices). It captured prescriptions issued in England and dispensed (fully filled and partly filled) in England, Wales, Scotland, Guernsey, Alderney, Jersey and the Isle of Man. However, it did not include prescriptions that were issued but not dispensed, so unfulfilled prescriptions were not accounted for. This is relevant in the context of the study of PERT shortage because unfulfilled prescriptions could help quantify the increases in workload, as many clinicians were providing multiple prescriptions to help patients access a supply. The secondary care dataset contained prescribing data from all NHS Trusts in England for medicines issued in NHS secondary care settings. As with primary care, it did not include private healthcare providers and NHS prescriptions supplied by hospitals but dispensed in community pharmacies.

Both datasets are updated monthly, and therefore, this study provided near real‐time trends up to February 2026. However, because secondary care data are finalized at the end of each financial year, data for secondary care were provisional from April 2025 to February 2026 and may be revised in the future. The revisions are not expected to alter the results of this study. The published datasets include detailed prescribing information across multiple administrative levels, from regions in England, and ICBs, down to individual GP practices. In this study, we focus on regional trends, with more granular analyses by ICB planned in the future. In addition, although the study period begins in January 2019, in the primary care dataset, the current seven‐region structure was introduced in April 2020 (replacing the previous four‐region structure, which could not be mapped to the new structure). Therefore, the regional analyses for primary care are from April 2020 onwards.

The SCMD dataset reports stock control data rather than individual patient prescriptions. This limits the conclusions that can be drawn from monthly medicine quantities alone. Capsule counts are influenced by several factors and may not directly reflect prescribing volume or trends in PERT use. For example, there is increasing recognition that many patients are underdosed, with starting doses often below guideline levels, and subsequent dose escalation required. Consequently, capsule counts may increase over time, and without the context of prescription numbers and quantities prescribed per prescription, these increases cannot necessarily be attributed to the shortage alone. However, in primary care, monthly prescription counts closely mirrored trends in capsule counts. This provides reassurance that the effects of the shortage were reflected consistently across both measures of prescribing volume, supporting the interpretation of the observed prescribing trends.

Furthermore, unlike primary care, which has well‐defined catchment populations, the populations served by secondary care providers are more difficult to define geographically. As a result, to calculate regional rates for secondary care, we used GP practice list sizes as the denominator, and there may be minor discrepancies between the true populations served by NHS trusts within each region and population estimates used in the analysis. Finally, both datasets do not include individual patient‐level information, so it was not possible to stratify prescribing by specific medical condition of pancreatic disorder or cystic fibrosis. However, this is not a limitation in terms of the aim and scope of the study, because the focus was on PERT shortage and overall prescribing trends.

### 4.3 Findings in the context of clinical management

The ongoing shortage continues to affect clinical practice and patients. Patient surveys reported reduced quality of life and difficulties accessing treatment,^16^ whereas media coverage highlighted the lengths to which patients went to obtain supplies.^28^ Early in the shortage, professional societies and patient organizations collaborated to produce clinical guidance for managing the PERT shortage. A position statement, issued by the Pancreatic Society of Great Britain and Ireland and endorsed by national charities, was published to support clinicians and patients.^15^ As the shortage progressed and supply chains adapted, this guidance evolved. Initial advice encouraged symptom management and maintenance of nutritional status using oral supplements (position statement v1). Subsequent versions prioritized smaller capsule formulations for paediatric patients and those with swallowing difficulties, and Creon Micro for infants (position statements v2–4). The sharp increase in Nutrizym and Pancrex prescribing reflected improving supply, but its use by clinicians unfamiliar with PERT alternatives contributed to new shortages, particularly of Pancrex powder, which is intended for enteral administration but was increasingly prescribed for oral use.^11^ This secondary shortage of this unique product was evident in the decline in secondary care prescribing towards the end of the study period.

The shortage also exposed regional differences in response and access to PERT. The absence of timely or coordinated national guidance, coupled with limited uptake of imported products, was particularly evident in London, where lower prescribing mirrored previously reported regional disparities in PERT prescribing.^29^ However, given the underrepresentation of London in primary care prescribing datasets,^30^ these findings should be interpreted with caution. Despite endorsement from the UK Government,^12^ the National Patient Safety Alert,^31^ the Specialist Pharmacy Service^32^ and individual ICBs,^26^, ^27^ our findings demonstrated minimal uptake of imported Pangrol. Responsibility for managing the shortage was largely delegated to ICBs, resulting in duplication of local guidance and variable implementation. Although most ICBs incorporated the Pancreatic Society's national recommendations,^15^ this fragmented approach represented an inefficient use of resources. Clinical and charitable organizations subsequently called for a coordinated national strategy,^33^ emphasizing the need for government intervention to facilitate imports, diversify the supplier base and safeguard patient access to essential medicines.

Clinically, PERT is complex to prescribe. Initiation is generally restricted to secondary care, and patients often require different preparations and doses at various times of the day. Over a period of time, with consistent use, prescriber support and subsequent relief of symptoms, patients learn to titrate the doses effectively according to factors such as meal type and size. This complexity, combined with the relatively low prevalence of PERT use (approximately 60 000 patients are prescribed PERT in the United Kingdom^14^), means that most primary care clinicians manage very few patients receiving PERT. Consequently, specialist expertise is limited, and switching between PERT formulations can be challenging.

Beyond compromising the management of PEI, PERT shortages may also create risks for clinically important drug–drug interactions by affecting the absorption of other orally administered medicines. This includes medicines that depend on adequate fat absorption, such as fat‐soluble vitamins, treatments for cystic fibrosis,^34^ hormonal contraception^35^ and other drugs affected by diarrhoea or malabsorption. Variable carbohydrate absorption may also contribute to glycaemic instability in patients with diabetes.^15^

From a policy perspective, our findings highlight the urgent need for a nationally coordinated contingency plan for essential medication shortages, including clear dose‐conversion guidance, therapeutic equivalence charts and real‐time supply alerts within electronic prescribing systems. The absence of real‐world pharmacovigilance data on the outcomes of substituted PERT products represents a gap requiring attention from regulatory bodies such as the MHRA and NICE. More broadly, the PERT shortage serves as a case study for strengthening medicine‐supply resilience across Europe, aligning with the WHO Essential Medicines Framework to ensure equitable and continuous access to critical treatments for PEI.

### 4.4 Future directions

This study provides foundational insights into prescribing behaviours in England during the PERT shortage. However, several areas warrant further investigation. First, patient‐level outcome studies are needed to evaluate the clinical consequences of disrupted PERT access, including weight loss, malnutrition‐related complications, poor glycaemic control and interruptions to cancer treatment and outcomes. Linking prescribing data with clinical outcomes could generate this important evidence. Second, qualitative research involving clinicians and patients is needed to provide a deeper understanding of experiences during the shortage, including perceptions of substitute therapies, decision‐making processes and adaptation strategies. Third, formal economic evaluations addressing both direct costs (e.g., drug costs) and indirect costs (e.g., staff time, additional training and cancelled or incomplete treatment courses) are needed to inform future resource planning.

Finally, implementation studies should assess the effectiveness of policy interventions introduced during the shortage, such as alternative product guidance, centralized procurement systems and communication strategies. Given evidence that PEI is underdiagnosed and PERT underprescribed in the United Kingdom, prescriber education remains a priority, with equal emphasis on non‐medical prescribers. PERT is an essential medicine that requires a high degree of prescriber‐patient interaction to achieve optimal outcomes. Suboptimal dosing may contribute to insufficient symptom relief, increasing complications and hospitalisations. Such evidence would strengthen future resilience planning and support continuity of care during future medicine shortages. Importantly, our findings also highlight the increasing national demand for PERT products in general, underscoring the need for proactive forward planning to ensure supply stability as demand continues to grow.

## 5 CONCLUSIONS

At the system level, our study exposed vulnerabilities in maintaining consistent access, including disrupted prescribing patterns, regional disparities and limited uptake of alternative and imported products. The PERT shortage has revealed critical gaps in care continuity for patients with PEI in England. This disruption demonstrated how the absence of an adequate contingency framework can lead to systemic failures, even for non‐acute but essential therapies. Policymakers, commissioners and clinical leaders must act urgently to enhance prescribing and supply‐chain resilience for essential medicines such as PERT. Safeguarding patient care during future shortages will require a coordinated national strategy, robust supply‐chain management, comprehensive prescriber support and rapid implementation of contingency policies. Proactive and unified responses will be essential to ensure continuity of therapy for patients dependent on PERT and other essential medicines.

## AUTHOR CONTRIBUTIONS

T.S.V., M.E.P., A.F., and A.L. conceived the study. T.S.V., L.F., C.W., and A.L. led the data analysis and prepared the first draft of the manuscript. All authors contributed to writing and revising the manuscript and approved the final version.

## CONFLICT OF INTEREST STATEMENT

M.E.P. has received honoraria for educational lectures and expert panel attendance from Viatris, PharmaNord, EssentialPharma and Nutricia Clinical Care. All other authors declare no conflicts of interest.

## Data Availability

The data that support the findings of this study are openly available from the NHS Business Services Authority (NHSBSA) https://www.nhsbsa.nhs.uk/prescription-data/prescribing-data. These data were derived from the following resources available in the public domain: English Prescribing Data (EPD), https://www.nhsbsa.nhs.uk/prescription-data/prescribing-data/english-prescribing-data-epd, and Secondary Care Medicines data, https://www.nhsbsa.nhs.uk/secondary-care-medicines-data.
